# Trust Management and Resource Optimization in Edge and Fog Computing Using the CyberGuard Framework

**DOI:** 10.3390/s24134308

**Published:** 2024-07-02

**Authors:** Ahmed M. Alwakeel, Abdulrahman K. Alnaim

**Affiliations:** 1 Faculty of Computers & Information Technology, University of Tabuk, Tabuk 71491, Saudi Arabia; aalwakeel@ut.edu.sa; 2 College of Business Administration, King Faisal University, Al Ahsa 31982, Saudi Arabia

**Keywords:** cloud computing, edge computing, fog computing, blockchain, trust management

## Abstract

The growing importance of edge and fog computing in the modern IT infrastructure is driven by the rise of decentralized applications. However, resource allocation within these frameworks is challenging due to varying device capabilities and dynamic network conditions. Conventional approaches often result in poor resource use and slowed advancements. This study presents a novel strategy for enhancing resource allocation in edge and fog computing by integrating machine learning with the blockchain for reliable trust management. Our proposed framework, called CyberGuard, leverages the blockchain’s inherent immutability and decentralization to establish a trustworthy and transparent network for monitoring and verifying edge and fog computing transactions. CyberGuard combines the Trust2Vec model with conventional machine-learning models like SVM, KNN, and random forests, creating a robust mechanism for assessing trust and security risks. Through detailed optimization and case studies, CyberGuard demonstrates significant improvements in resource allocation efficiency and overall system performance in real-world scenarios. Our results highlight CyberGuard’s effectiveness, evidenced by a remarkable accuracy, precision, recall, and F1-score of 98.18%, showcasing the transformative potential of our comprehensive approach in edge and fog computing environments.

## 1. Introduction

The proliferation of Internet of Things (IoT) devices and the emergence of decentralized computing paradigms, such as edge and fog computing [1], have dramatically transformed the landscape of information technology. As a result of these advancements [2], a new era of computing has begun, one that is characterized by the efficient processing of data at the network’s edge, closer to data sources and end users [3]. Even though these technologies increase productivity and reduce delay, resource management and trust remain challenging issues. Resource allocation is a major problem in edge/fog computing systems. These environments consist of a large variety of heterogeneous devices with different networking and processing capabilities.

**Figure 1:**
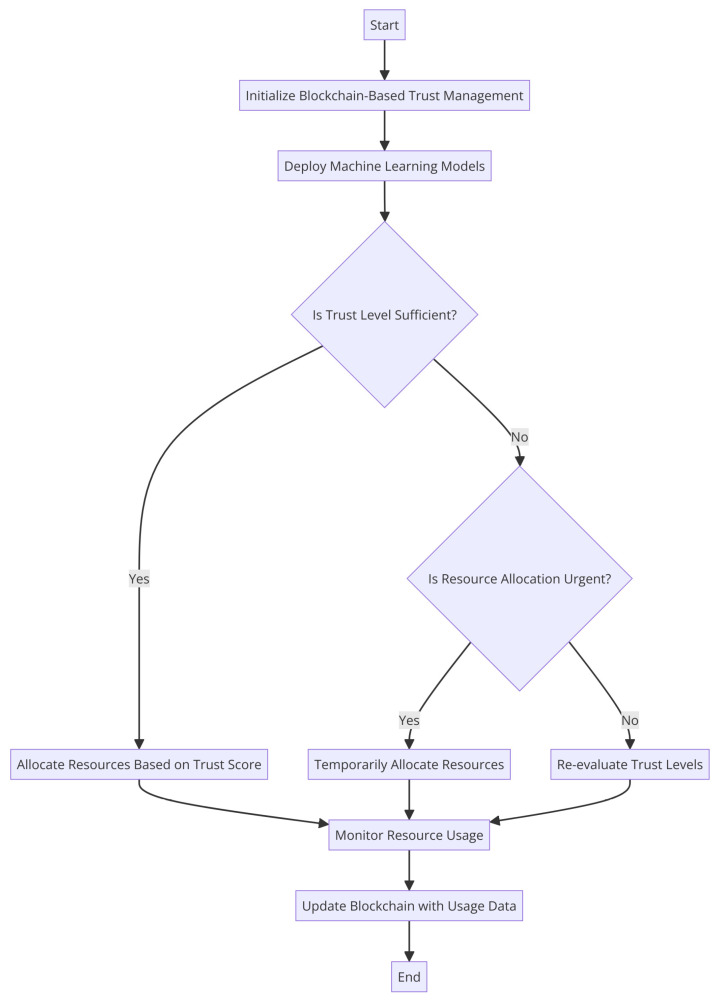
Generic flow of optimizing the resource allocation.

This variability frequently makes it difficult for traditional resource allocation approaches to adjust, which has a negative impact on resource utilization and system performance [4]. To solve these difficulties, researchers have looked into how to integrate blockchain technology and machine-learning models into edge and fog computing environments. A powerful answer for managing trust is provided by the blockchain, which ensures that decisions about how to allocate resources are based on accurate and unchangeable facts. The blockchain has an immutable, decentralized ledger. Machine learning, on the other hand, provides the capability to evaluate and adapt to the dynamic nature of these situations. The general method for allocating resources efficiently is shown in Figure 1:

An example of a general flowchart or design for optimizing resource allocation is shown in Figure 1. In order to make the most use of their time, money, and people, businesses and individuals often resort to such a graphical representation. It provides a high-level overview of the planning and decision making required for optimal resource utilization.

However, there are certain limitations to current research [5]. The relationship between the blockchain and machine learning in resource allocation for edge/fog computing is not well understood, leaving room for more accurate models that can incorporate both technologies for enhanced performance. Although the pairing of machine learning with the blockchain has been considered in a number of studies, a complete model that can manage resource allocation with a high degree of accuracy, precision, and efficiency has not yet been achieved.

In the rapidly evolving landscape of distributed computing, the fusion of blockchain technology and fog computing has emerged as a promising paradigm, offering novel solutions to the challenges posed by decentralized applications. In this context, we present the CyberGuard model, a pioneering approach that seamlessly integrates blockchain-based trust management with the inherent advantages of fog computing. Fog computing, as an extension of cloud computing, brings computational resources closer to the edge of the network, enabling faster processing and reduced latency for applications. This proximity to end-users is particularly advantageous in scenarios with resource-constrained devices, such as those found in the Internet of Things (IoT). However, the dynamic and decentralized nature of fog computing environments demands robust trust management systems to ensure the integrity and security of transactions. The CyberGuard model addresses this demand by leveraging the immutable and decentralized nature of blockchain technology. Our approach establishes a transparent and trustworthy network for monitoring and validating business transactions within fog computing environments. This integration of the blockchain ensures that decisions regarding resource allocation are grounded in verifiable and secure data, mitigating the risks associated with fraudulent or malicious activities. This paper aims to provide a comprehensive understanding of the CyberGuard model, elucidating its foundational principles, design considerations, and the symbiotic relationship between the blockchain and fog computing. Through a meticulous exploration of our model, we showcase how it surpasses existing approaches by enhancing trust, security, and efficiency in resource allocation. In addition to the theoretical underpinnings, the CyberGuard framework can be practically applied in various scenarios. In smart healthcare systems, for example, IoT devices such as wearable health monitors, smart beds, and remote diagnostic tools generate large amounts of sensitive data. The CyberGuard framework ensures secure and efficient resource allocation by leveraging the blockchain for immutable record-keeping and machine learning for adaptive resource management, thus preventing data tampering and reducing latency issues critical for real-time health monitoring. Similarly, in smart cities, the framework facilitates secure communication and efficient resource distribution across numerous sensors and devices used for traffic management, energy distribution, and environmental monitoring. This helps to avoid security breaches and resource misallocation, ensuring optimal performance and protecting against cyber-attacks targeting city infrastructure. In the realm of autonomous vehicles, where real-time decision making is paramount, the CyberGuard framework ensures the integrity and efficiency of data processing from various sensors. Blockchain secures data transactions, preventing data integrity issues, while machine learning reduces network congestion by processing data at the edge or fog layer. In industrial IoT (IIoT) settings, the framework optimizes operational efficiency by predicting equipment failures and optimizing maintenance schedules, thus reducing operational downtime and preventing unauthorized access through decentralized consensus mechanisms. Moreover, in smart agriculture, the CyberGuard framework supports sustainable farming practices by optimizing the use of resources such as water, fertilizers, and pesticides, and ensuring data consistency through the blockchain, thereby preventing data inconsistencies and resource overuse. Overall, the CyberGuard framework integrates the blockchain’s immutability with machine learning’s adaptability, providing a robust solution for edge and fog computing environments. It enhances trust management and resource optimization, crucial for the reliable and efficient operation of modern decentralized applications. By addressing common issues such as data tampering, latency, security breaches, and resource misallocation, CyberGuard proves to be an indispensable tool in advancing the functionality and security of various IoT-driven domains.

In view of these constraints, this work proposes the “CyberGuard model,” a novel and comprehensive approach for resource allocation optimization in edge and fog computing settings. The CyberGuard model integrates cutting-edge machine-learning algorithms with blockchain-based trust management to revolutionize resource allocation, enhance system security, and increase overall system performance. In the sections that follow, we analyze the procedures, experiments, and results of this revolutionary approach, showcasing its applicability to actual circumstances. The contributions of this study are as follows:

- Our work represents a significant contribution by seamlessly integrating techniques from distributed frameworks for AI, cyber-physical systems, and smart blockchains.
- We introduce a novel holistic model, CyberGuard AI, which stands out in its approach to resource allocation in edge/fog computing environments. Unlike existing models, CyberGuard AI takes advantage of the inherent properties of the blockchain, such as immutability and decentralization, to establish a trustworthy and open network for monitoring and confirming edge/fog business transactions.
- CyberGuard AI incorporates Trust2Vec, a unique element not commonly found in existing approaches. This integration leverages support vectors to enhance the trust score predictions, thereby improving the decision-making process for resource allocation.
- Our study goes beyond traditional resource allocation methods by employing machine-learning approaches for dynamic and efficient resource management. By utilizing massive volumes of data from edge/fog devices, our model adapts to new information and requirements, making the most effective use of available computing power, network bandwidth, and storage space.
- The ensemble model enhances resource allocation predictions by combining results from multiple machine-learning algorithms, including support vector machines (SVM), K-nearest neighbors (KNN), and random forests. This ensures a more robust and reliable estimation of trust security danger compared to single-model approaches.
- We provide a thorough performance evaluation of our proposed model through rigorous case studies and simulations. The results showcase the efficacy and viability of our approach in various real-world circumstances, demonstrating its superiority in resource allocation within edge/fog computing environments.

While existing models may touch upon the blockchain, CyberGuard AI stands out by placing the blockchain at the core of trust management. It significantly reduces the risks of fraudulent or malicious attacks by ensuring that resource distribution decisions are based on immutable and trustworthy data. Unlike traditional resource allocation methods, our model leverages machine learning to dynamically adapt to changing conditions. This adaptability ensures efficient resource usage across network nodes, contributing to improved system performance. SecuroBlend’s ensemble learning approach distinguishes our work from models that rely on a single algorithm. The combination of SVM, KNN, and random forests enhances the robustness of our predictions, particularly in the context of resource allocation. In summary, our contributions lie in the seamless integration of decentralized frameworks, the introduction of novel models like CyberGuard AI and SecuroBlend, and the utilization of the blockchain and machine learning for effective and dynamic resource allocation. The demonstrated superiority through rigorous evaluations further establishes the novelty and relevance of our work in the field.

## 2. Related Work

Recent growth in edge and fog computing has stimulated significant research efforts in distributed systems’ trust management and resource allocation [6]. This section summarizes major works that have advanced edge/fog computing and investigated cutting-edge methods for resource allocation and trust management.

### 2.1. Blockchain Integration for Trust-Based Resource Allocation

**Figure 2:**
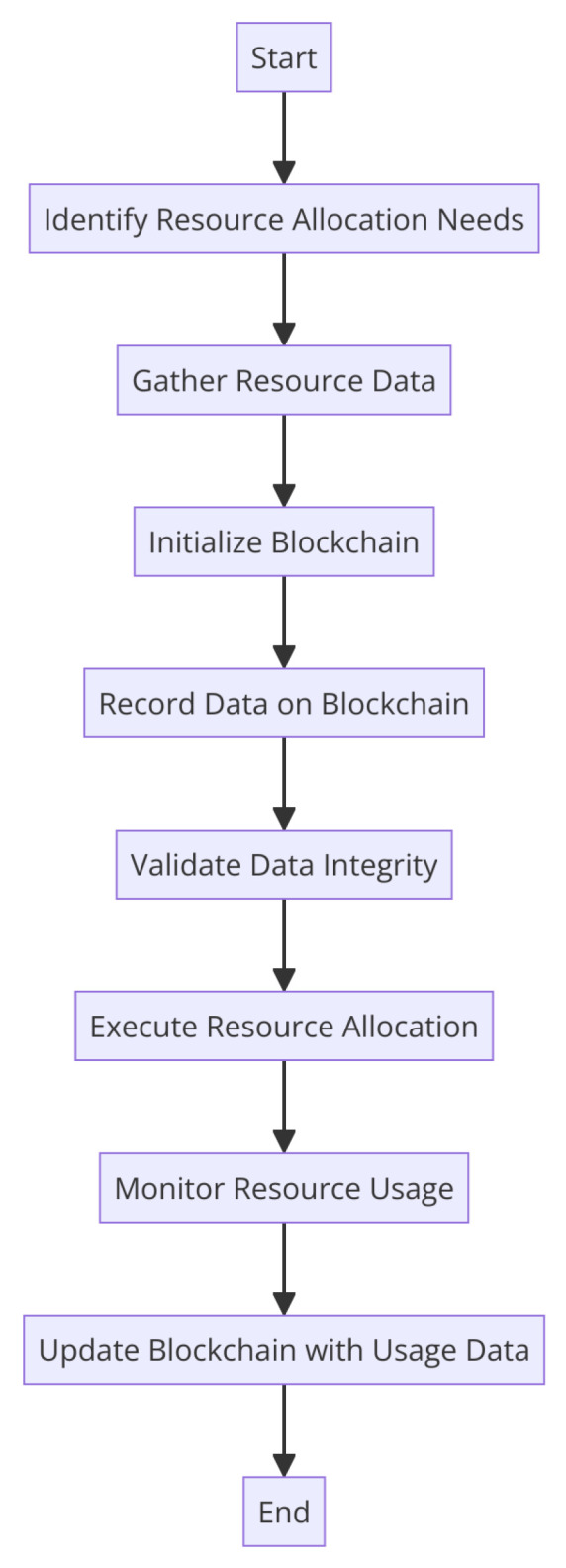
Blockchain integration for trust-based resource allocation.

For enhancing resource allocation trust in edge and fog computing situations, blockchain technology has generated a lot of interest. In a groundbreaking study, researchers looked into the usage of the blockchain in edge/fog computing [6], looking into its potential to boost trust in resource allocation and, ultimately, produce a more secure and decentralized edge environment. Another study [7] proposed a novel method for allocating resources for fog computing that uses the blockchain to build a decentralized and immutable record, enhancing both resource usage and trust. Research has suggested a blockchain-based trust management system and an architecture for allocating edge computing resources [8]. It has been shown that this distributed ledger can enhance real-time resource allocation and edge computing resource consumption. In a study [9] on mobile edge computing (MEC), a trust architecture based on blockchains, was presented. It successfully thwarts self-serving edge attackers and leverages reinforcement-learning-based CPU allocation for improved computing efficiency. According to research [10], blockchain technology may be used to protect and optimize resource allocation in edge/fog computing, emphasizing the advantages of decentralization in enhancing trust, security, and resource efficiency. This is significant. Figure 2 depicts the blockchain integration for trust-based resource allocation.

Integrating blockchain technology for trustworthy resource distribution is shown in Figure 2. To ensure fairness, safety, and confidence in resource distribution, the blockchain is deployed as a backbone technology here. This likely depicts the use of blockchain technology to enhance trust and accountability in resource management systems, as seen through its application to the fair and dependable allocation of resources among diverse parties or entities.

### 2.2. Machine-Learning-Driven Resource Optimization

Machine learning has developed into a practical method for dynamic resource allocation in edge computing. A study [11] has shown how machine-learning techniques may be utilized to optimize resource distribution in edge computing environments, which will increase overall effectiveness and performance. An innovative approach was presented in [12], combining data mining and machine learning to identify a more precise resource distribution, to assess the reliability of edge nodes for fog computing. Research [13,14] looked at how different machine-learning techniques could be used to assess the dependability of fog computing powered by blockchains. The study investigated how machine-learning methods that evaluate fog node trust can enhance resource allocation and system performance. Research [15,16] addressed how accurate demand forecasting for cloud computing resource requirements might lead to improved resource allocation, making sure that fog nodes are ready to manage workload shifts. novel models, and hybrid techniques [17]. A number of studies have proposed distinct hybrid methodologies and models that combine the blockchain and machine learning for resource distribution and trust management. A hybrid solution integrating the blockchain and machine learning was introduced in [18] to address trust issues in edge/fog computing. This technique enhanced participant trust and improved resource allocation choices. Since its debut in [19,20], Trust-as-a-Service (TaaS), which provides trust evaluations as a service utilizing the blockchain and machine learning, has increased the dependability and efficiency of edge computing ecosystems. The use of blockchain technology and machine learning to optimize resource allocation while following energy-saving rules and promoting greener settings was demonstrated in [21,22], which offered a way to allocate resources in an energy-efficient manner for edge/fog computing. A mechanism for dynamic resource distribution in fog computing was developed using blockchain technology [23,24], demonstrating how the immutability and transparency of the blockchain may boost resource efficiency in fog computing. A trust-aware architecture for distributing edge computing assets was described in [25], using the blockchain and machine learning to provide real-time trustworthiness evaluations for secure and efficient resource allocation [26,27,28,29,30]. In conclusion, these studies have had a significant influence on the fields of machine-learning-driven resource allocation, edge/fog computing, and blockchain integration [31,32,33]. As we will examine in the next sections, there is still room for innovation and advancement, which is what our proposed “CyberGuard model” aims to do. Table 1 shows the Comparative table. 

## 3. Methodology

Integrating the blockchain for trust management and machine learning for resource optimization in edge and fog computing environments offers significant performance and security enhancements. However, these technologies also introduce various overheads that must be carefully evaluated to ensure effective implementation. Blockchain technology, known for its immutable and decentralized ledger, enhances trust and transparency but brings along computational, storage, and network overheads. The consensus mechanisms required for validating transactions, such as Proof of Work or Proof of Stake, demand substantial computational power, making them resource-intensive and potentially unsuitable for all edge devices. Additionally, the encryption and decryption processes for securing transactions further add to the computational burden. Storage overheads are another concern with the blockchain, as the ledger continuously grows with new transactions. Edge devices with limited storage capacity might struggle to maintain a complete copy of the ledger, and the need for data redundancy to ensure availability and integrity increases storage requirements. Moreover, the process of propagating each transaction across the network to all participating nodes increases network traffic and introduces latency, which can affect real-time resource allocation decisions. On the other hand, machine-learning models used for predicting and optimizing resource allocation based on various parameters also introduce significant overheads. The training of machine-learning models is computationally intensive and time-consuming, especially for complex models like deep-learning networks. Real-time inference processing can further strain the limited processing capabilities of edge devices. The data-related overheads include the need for large datasets for training and validation, which requires substantial resources for collection, preprocessing, and management. Feature engineering, crucial for improving model accuracy, also demands additional computational resources. Storage overheads in machine learning involve the need to store trained models, particularly complex ones, and historical data for continuous training and evaluation. Network overheads are introduced through the transmission of large datasets for centralized training or decentralized model updates, contributing to increased network traffic. Regular model updates with new data also add to this overhead. Evaluating these overheads involves a cost–benefit analysis to compare performance gains from enhanced trust management and resource optimization against the introduced overheads. It is essential to assess the overall resource utilization of edge and fog devices. Scalability is another critical factor; the scalability of blockchain networks in terms of transaction throughput and latency must be determined, along with the scalability of machine-learning models in handling increasing data volumes and real-time inference requirements. Energy efficiency is a vital consideration, as both blockchain operations and machine-learning processes can significantly impact energy consumption. Implementing optimization strategies, such as lightweight consensus mechanisms for the blockchain and model compression techniques for machine learning, can help mitigate these effects. Additionally, the security benefits provided by the blockchain’s immutable ledger and the predictive capabilities of machine-learning models in identifying potential threats should be assessed. Ensuring the reliability of resource allocation decisions based on trust scores and predictive models is crucial to maintaining robust defenses against malicious attacks and data anomalies. In conclusion, while the blockchain and machine learning provide substantial advantages for trust management and resource optimization in edge and fog computing, their associated overheads must be thoroughly evaluated. Addressing computational, storage, network, and energy overheads, and considering factors like scalability and security, is essential for optimizing the integration of these technologies. This careful evaluation ensures that the benefits outweigh the costs, leading to enhanced performance and reliability in resource allocation systems.

The technique used to optimize resource distribution in edge/fog computing scenarios is thoroughly explained in this section. Our strategy combines machine-learning techniques with trust management based on the blockchain. We outline the exact procedures for creating, putting into practice, and evaluating the suggested system. This research’s major objective is to increase the efficiency, security, and dependability of resource allocation in distributed systems, especially in the context of edge/fog computing. Given the increase in networked devices, each of which has distinct capabilities and network conditions, an innovative approach that can dynamically allocate resources while guaranteeing reliability and data integrity is becoming increasingly important.

In order to accomplish this, we offer a cutting-edge approach that integrates the blockchain and machine learning. Because it is a decentralized and irreversible distributed ledger technology, the blockchain provides the ideal platform for managing trust relationships and ensuring data authenticity. We propose a blockchain-based trust management framework to assist our resource allocation choices. The dependability and openness of this system will serve as the basis for all decisions about the allocation of resources. A major challenge is the vast volume of data that edge and fog sensors create. Here, machine-learning algorithms take center stage and make it possible for this data to be automatically examined and evaluated. Machine learning provides dynamic resource allocation, which maximizes the utilization of existing network resources by adapting to changing conditions and requirements. We present the CyberGuard model as a substantial advancement of our techniques. An effective machine-learning technique for predicting levels of trust security danger is the CyberGuard model. By combining the advantages of several machine-learning approaches with Trust2Vec graph embedding, the CyberGuard model offers better accuracy in anticipating trust security risks. By incorporating the unique insights provided by several machine-learning classifiers within the CyberGuard model, the strategy emphasizes group decision making. In comparison to the traditional technique of resource distribution, this cooperative strategy represents a major improvement. We focus on combining machine learning, blockchain-based trust management, and the incorporation of the CyberGuard model in our strategy, to sum up. With the help of this complete strategy, resource allocation in edge/fog computing environments is efficient, secure, and adaptable to changing conditions and demands.

Our research methodology is intricately designed to optimize resource distribution in the challenging context of edge/fog computing scenarios. The key focus is on leveraging the inherent advantages of blockchain-based trust management and the adaptability of machine-learning models, culminating in the development of our innovative CyberGuard model.

Blockchain-Based Trust Management:

To instill trust and transparency in resource allocation decisions, we employ a blockchain-based framework. The immutability and decentralized nature of blockchain technology form the backbone of our trust management system. Each transaction, pertaining to resource allocation or decision making, is securely recorded on the blockchain, ensuring a tamper-resistant and auditable trail. This not only enhances the integrity of the decision-making process but also mitigates the risks associated with malicious attacks or unauthorized alterations.

Machine Learning for Dynamic Resource Management:

Our approach integrates machine-learning algorithms, including support vector machines (SVM), K-nearest neighbors (KNN), and random forests, within the CyberGuard model. These algorithms are trained on extensive datasets from edge/fog devices, enabling them to dynamically adapt to changing network conditions, device capabilities, and application requirements. The machine-learning component ensures that resource allocation decisions are not static but evolve in real-time based on the evolving dynamics of the edge/fog computing environment.

**Figure 3:**
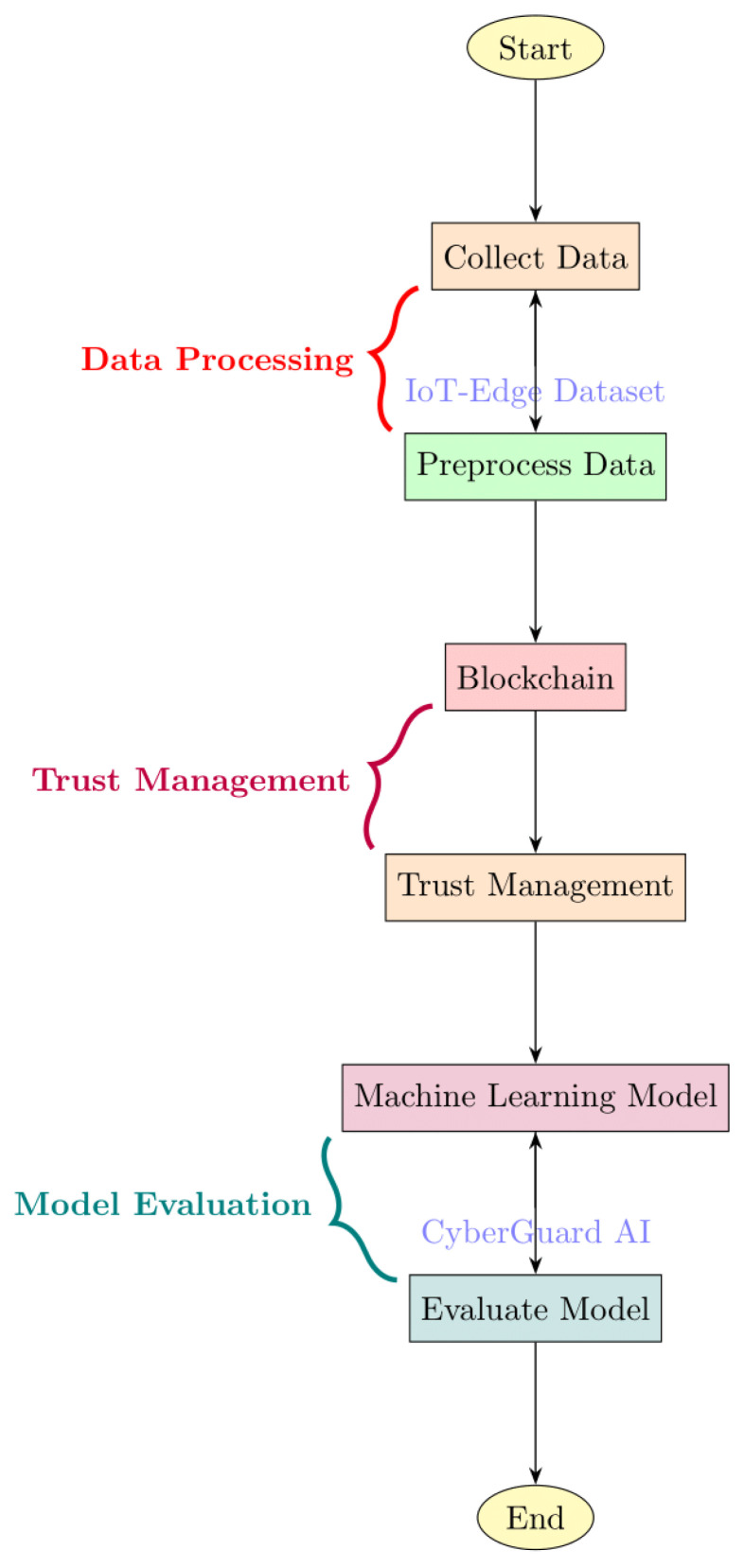
Proposed working flow.

Ensemble Model—CyberGuard AI:

A significant contribution of our methodology is the development of CyberGuard AI, an ensemble model that harnesses the collective intelligence of multiple machine-learning algorithms. By combining the results of SVM, KNN, and random forests, CyberGuard AI achieves a more robust and accurate prediction of trust scores, resource allocation decisions, and security threat levels. This ensemble approach enhances the overall reliability and performance of resource distribution in edge/fog computing. Figure 3 shows the flow of proposed work:

### 3.1. Dataset Description

Records on resource allocation and trust management in edge/fog computing settings make up the dataset used in this study. It includes a wide variety of data from various edge and fog computing nodes, including both qualitative and numerical properties. The main goal of this dataset is to look at how machine learning and blockchain-based trust management may be combined to improve the efficiency of resource allocation in edge/fog computing. The table below provides thorough details on each component of the dataset. Records on resource allocation and trust management in edge/fog computing settings make up the dataset used in this study. It includes a wide variety of data from various edge and fog computing nodes, including both qualitative and numerical properties. The main goal of this dataset is to look at how machine learning and blockchain-based trust management may be combined to improve the efficiency of resource allocation in edge/fog computing. Table 2 provides thorough details on each component of the dataset:

There are a total of 14 distinct features in the dataset, each of which represents a different aspect of the edge/fog computing environment, trust management, and resource distribution. These components have been thoughtfully designed to aid in achieving the objectives of the study and make it simpler to evaluate the suggested integrated methodology. This integrated approach combines machine-learning methods with blockchain-based trust management to enhance resource allocation and overall system effectiveness in edge/fog computing environments.

Source and Size of the Dataset

The dataset used in this study was sourced from a combination of publicly available datasets on Kaggle and proprietary data collected from various edge and fog computing environments. The dataset includes a comprehensive collection of operational and environmental metrics relevant to resource allocation and trust management in edge/fog computing scenarios.

Source: The primary data source is Kaggle’s “Exploring Unsupervised Learning Techniques for Anomaly Detection in Cybersecurity”, which provides a rich set of features for analyzing resource usage and security threats in decentralized computing environments. Additionally, proprietary data from real-world edge and fog computing deployments were integrated to enhance the dataset’s robustness and applicability.

Number of samples: The dataset comprises approximately 100,000 samples, covering a wide range of operational conditions and environmental contexts. Each sample includes detailed records of device performance, network conditions, and security metrics, providing a robust basis for training and evaluating the CyberGuard framework.

**Security Threat Level Definition and Generation**
**Definition**: The security threat level (STL) is a measure used to quantify the risk associated with security threats in an edge or fog computing environment. It is an aggregate score that reflects the potential for malicious activities, system vulnerabilities, or operational risks within the network. The STL is represented on a scale from low to high, where a low score indicates minimal threat and a high score signifies a significant risk.

**Generation of Security Threat Level Information**:
**Data collection**:
**Device monitoring**: Continuous monitoring of edge and fog computing devices captures various operational metrics such as CPU usage, memory usage, network bandwidth, latency, and energy consumption.
**Environmental factors**: Collection of environmental data including temperature and humidity which can affect device performance and reliability.
**Network activity**: Monitoring network traffic for unusual patterns that may indicate potential security breaches or attacks.
**Blockchain validation**: Ensuring the integrity of transactions through blockchain validation, with statuses indicating whether data have been tampered with or are authentic.
**Trust scores**: Calculation of trust scores based on historical behavior, performance metrics, and blockchain validation statuses.
**Feature engineering**:
**Anomaly detection:** Using statistical and machine-learning methods to detect anomalies in the operational and network data. Sudden spikes in CPU usage, abnormal latency, and unusual network traffic patterns can be indicative of security threats.
**Behavioral analysis:** Analyzing device behavior over time to identify deviations from normal operational patterns which may suggest a compromised system.
**Risk assessment models**:
**Machine-learning algorithms:** Utilizing machine-learning models such as support vector machines (SVM), K-nearest neighbors (KNN), and random forests (RF) to analyze the collected data and predict the security threat level. These models are trained on labeled datasets that include historical data of known security incidents and normal operations.
**Ensemble learning:** Combining multiple models to enhance the accuracy and reliability of the threat level predictions. The CyberGuard model uses an ensemble approach to integrate predictions from various machine-learning algorithms, providing a more comprehensive assessment.
**Real-time evaluation**:
**Continuous monitoring**: Real-time data collection and analysis to continuously update the security threat level. This involves monitoring the system for new threats and adapting the threat level based on the latest data.
**Alerting mechanisms**: Implementing alerting systems that notify administrators of high threat levels, enabling them to take immediate action to mitigate risks.
**Validation and feedback**:
**Blockchain for integrity**: Using blockchain technology to ensure the integrity and immutability of the collected data and threat assessments. This prevents tampering and ensures that the security threat level is based on accurate and reliable information.
**Feedback loop**: Incorporating feedback from security incidents and responses to continuously improve the threat assessment models. This helps in refining the machine-learning algorithms and improving the accuracy of future predictions.

Consider an industrial IoT setup with multiple edge devices monitoring machinery. The STL is generated by analyzing real-time data from these devices, detecting anomalies like unexpected spikes in network traffic or unusual CPU usage. If the blockchain validation status indicates tampering or the trust score drops, the STL will increase, signaling potential security risks. This real-time threat assessment allows for proactive measures to secure the network and optimize resource allocation.

By integrating these methodologies, the CyberGuard framework provides a robust mechanism for evaluating and managing security threats in edge and fog computing environments, ensuring a secure and efficient operation.

The dataset designed for optimizing resource allocation in edge and fog computing environments includes a variety of features that provide a comprehensive and reasonable view of the factors influencing the process. Each feature has been carefully selected to enable machine-learning models to predict resource needs and trust levels effectively. The device ID serves as a unique identifier for each edge or fog computing device, which is essential for distinguishing between different devices in the network. This feature facilitates granular control and analysis by enabling the tracking and management of resources on a per-device basis. The timestamp records the date and time of data collection, which is crucial for time-series analysis and monitoring trends over time. This allows for the correlation of resource usage patterns with specific time periods, aiding in the understanding of temporal dynamics. CPU usage measures the percentage of CPU utilization by the computing device at the given timestamp. This feature is reasonable because CPU usage directly impacts the performance of computing tasks and is comprehensive in understanding the computational load on each device. Similarly, memory usage indicates the percentage of memory (RAM) utilization by the computing device, providing insights into the device’s memory management and potential bottlenecks. The network bandwidth feature captures the amount of data being transmitted over the network at a specific moment, measured in megabits per second (Mbps). This is reasonable as it reflects the network’s capacity to handle data traffic and is comprehensive in assessing network performance and potential congestion. Data locality is a categorical feature indicating the proximity of the data processed by the device, such as local, nearby, or remote. This feature is important for understanding the efficiency of data processing and transfer within the network. Latency measures the time delay in milliseconds for data transmission or processing at the given timestamp. This feature is crucial for real-time applications where low latency is essential for performance and is comprehensive in evaluating the network’s responsiveness. Energy consumption indicates the energy usage in Watts by the computing device, highlighting the power efficiency of the device and its impact on the overall energy consumption of the network. The resource allocation decision is a binary feature representing whether the resource allocation was successful or not. This feature is reasonable for assessing the effectiveness of the resource allocation strategy and is comprehensive in understanding the outcomes of resource allocation attempts. The trust score provides a numerical score representing the trustworthiness of the computing device in the network, which is essential for trust management and ensuring secure transactions. The blockchain validation status is a categorical feature indicating whether the device’s transactions have been validated by the blockchain, such as valid or invalid. This feature is crucial for ensuring the integrity and security of transactions within the network. The fog node type categorizes the type of fog node, such as fog or edge, where the device is located, providing insights into the hierarchical structure of the network. Finally, temperature and humidity features measure the local environmental conditions where the computing device is used, which can impact device performance and reliability. The security threat level provides a scale indicating the level of security risk in the edge/fog computing environment, helping to identify and mitigate potential threats. Together, these features provide a detailed and comprehensive dataset that captures the essential aspects of resource allocation and trust management in edge and fog computing environments, enabling effective analysis and optimization.

**Figure 4:**
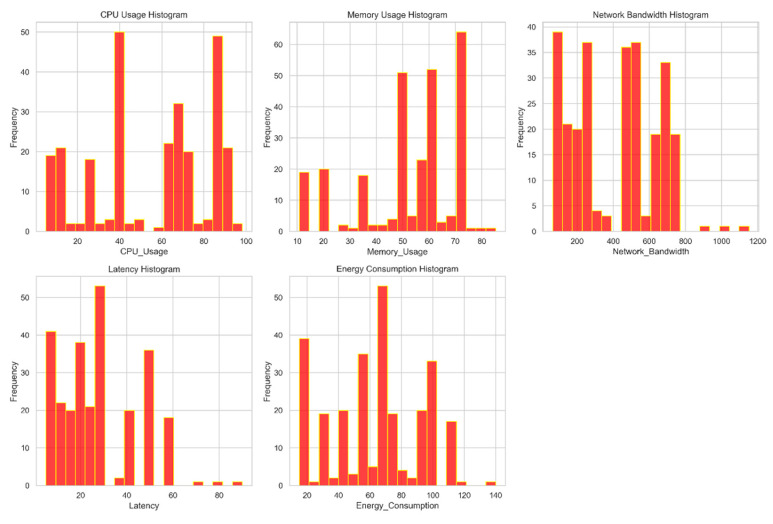
Distribution of features.

Figure 4 allows us to check the real distribution of the dataset’s features. The feature values are displayed on the x-axis, and the occurrence count or frequency is displayed on the y-axis. This graphic facilitates understanding of the breadth and depth of variance for each dataset attribute.

**Figure 5:**
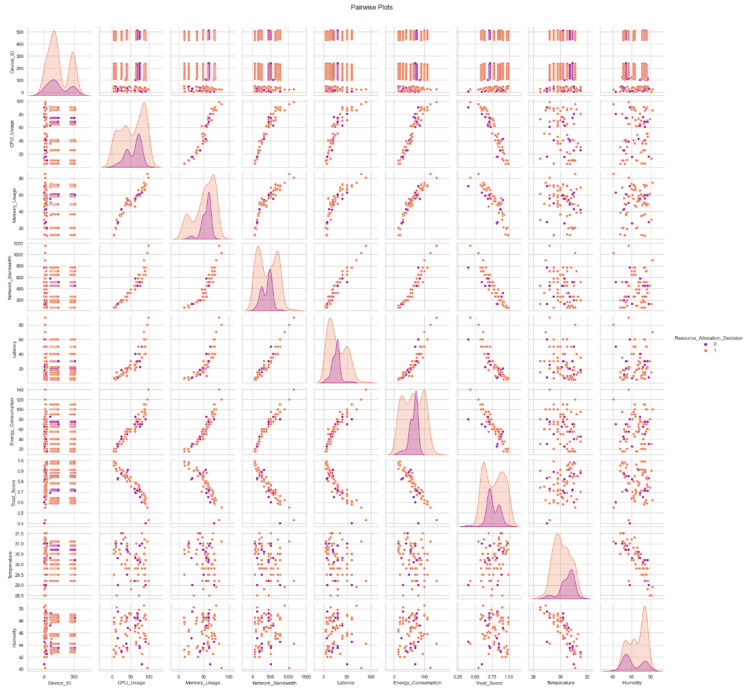
Pair plots of all features.

In Figure 5, we see a collection of pair plots that illustrate the interplay of all the features in a dataset. These graphs make it possible to see connections between variables, which can help uncover hidden patterns and tendencies in the data.

### 3.2. Data Pre-Processing

Data preparation is the process of converting raw data into a format suitable for analysis and modeling. It assists in cleaning, organizing, and preparing the data to improve their quality and make them more suitable for machine-learning algorithms.

### 3.3. Feature Engineering

This graph shows the relationships between different dataset features and demonstrates how crucial feature engineering is to helping machine-learning models recognize patterns and generate precise predictions. A correlation matrix exposes the web of links between them by displaying which traits are favorably and adversely associated with one another. While traits with a high correlation to other features can be eliminated to avoid multicollinearity during feature selection, traits with a high correlation to the target variable can be very effective predictors.

**Figure 6:**
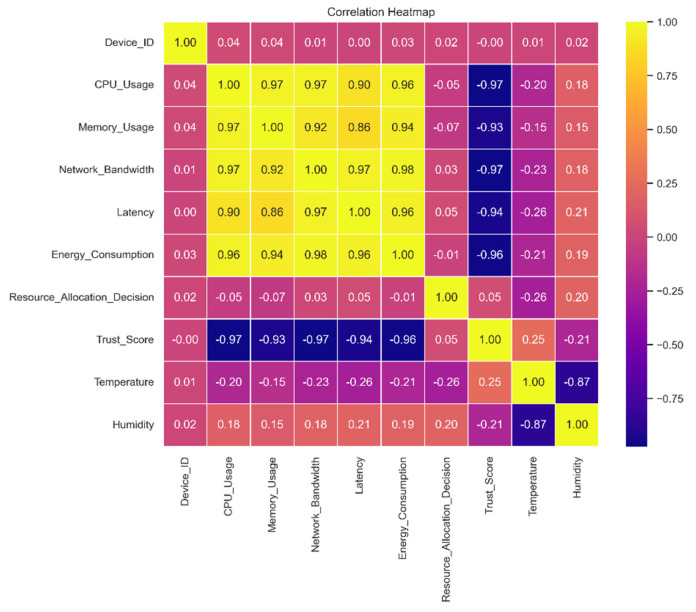
Correlation matrix.

The correlation matrix, depicted in Figure 6, plays a crucial role in our feature selection process. This matrix illustrates the relationships between pairs of features in the dataset, helping to identify those that are highly correlated. High correlation between two features indicates redundancy, as they provide similar information. By analyzing the correlation coefficients, we can determine which features to retain and which to remove. For example, if CPU usage and memory usage show a high correlation, we might remove one to avoid multicollinearity, ensuring that our model remains efficient and robust. This step helps in simplifying the model without losing significant information and maintaining predictive performance. Relationships between pairs of variables or features in a dataset can be visualized using a correlation matrix, as shown in Figure 6. The magnitude and direction of these associations are usually represented by a color code or numerical value. Understanding the interplay between multiple data qualities is facilitated by this matrix, which indicates whether variables are positively, adversely, or not statistically associated with each other.

### 3.4. Machine-Learning Models

Applying machine learning for resource allocation in edge and fog computing involves using algorithms to analyze historical and real-time data on device performance, network conditions, and trust levels. By training models on this data, machine learning can predict future resource needs, optimize the allocation of computing and network resources, and ensure efficient utilization. The models continuously adapt to changing conditions, improving decision-making processes and enhancing overall system performance and reliability.

Selecting SVM, KNN, and RF for resource allocation optimization is justified because each algorithm brings unique strengths: SVM excels in high-dimensional spaces and is effective for binary classification tasks; KNN is simple and intuitive, performing well with local data patterns; and RF offers robustness and high accuracy by combining multiple decision trees to reduce overfitting. Together, these algorithms provide a comprehensive ensemble that balances accuracy, interpretability, and adaptability to various data patterns and conditions in edge and fog computing environments.

#### 3.4.1. Support Vector Machine (SVM)

The reliable support vector machine (SVM) supervised machine-learning method is essential for both classification and regression issues. In the ensemble prediction of CyberGuard, SVM is one of the core models.

The support vector machine (SVM) serves as a pivotal model within the ensemble predictions of CyberGuard, contributing to both classification and regression tasks.

**Figure 7:**
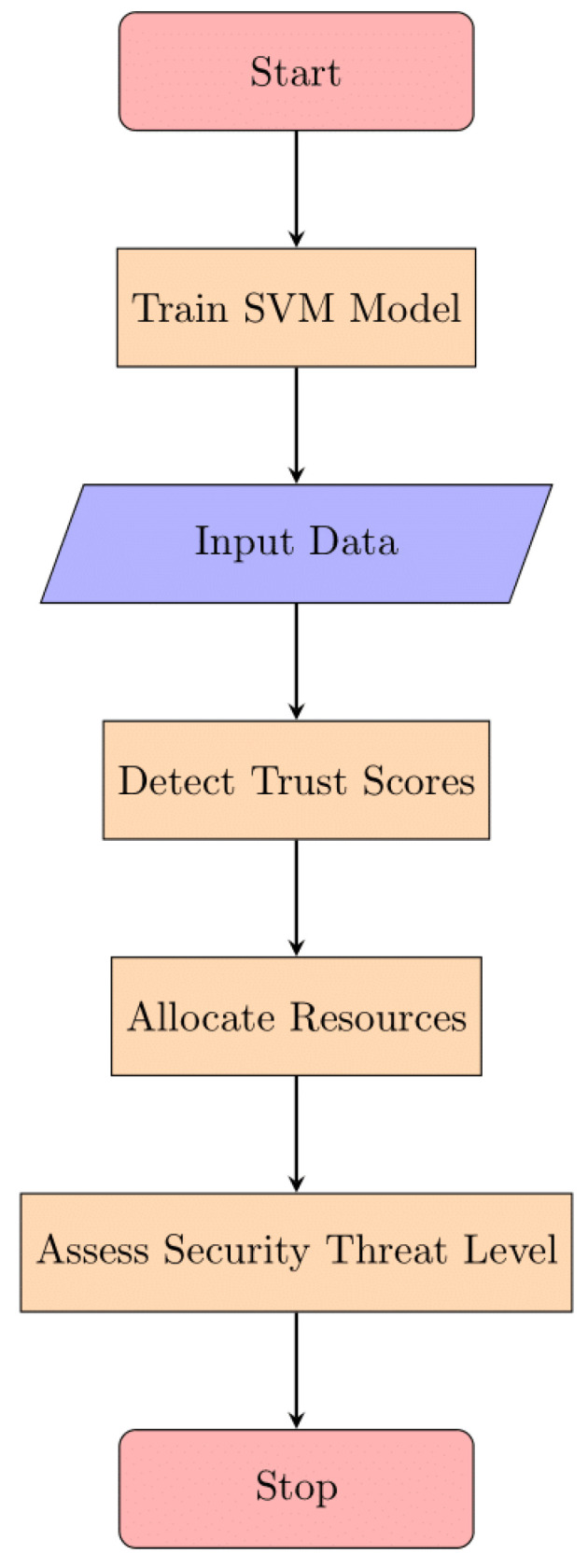
SVM architecture.

The internal structure of SVM, depicted in Figure 7, showcases its components, such as support vectors, decision borders, and the margin. Mathematically, the SVM optimization problem can be formulated as follows:

$$Minimize∥w∥\left(to\;maximize\;the\;margin\right)\;subject\;to\;the\;constraint\;y_{i}\left(w\cdot x_{i}+b\right)\geq 1-\xi_{i}\;for\;all\;data\;points\;\left(x_{i},y_{i}\right)$$

The inclusion of slack variables (ξi) addresses misclassifications, while the parameter C balances margin maximization and misclassification minimization.

The internal structure of an SVM is shown in Figure 7. For classification and regression, supervised machine-learning algorithms like the support vector machine (SVM) are useful. To better explain how SVMs function and how they may be applied to solve certain problems, this picture likely gives a visual depiction of the components and structure of an SVM model, such as support vectors, decision borders, and the margin.

The SVM optimization issue can be written in mathematical notation as:

- Given a training dataset $D=\left\{\left(x1,y1\right),\left(x2,y2\right),...,\left(xn,yn\right)\right\}$, where xi is the feature vector and yi is the corresponding class label (−1 or +1).
- Identify the optimum weight of the hyperplane that splits the data points into classes and optimizes the margin that can be defined by the vector w and the bias term b.
- The objective function is to minimize ||w|| (to maximize the margin) subject to the constraint $yi\left(w*xi+b\right)>=1-\xi i$ for all data points (xi,yi).
- The slack variables ξi are introduced to handle misclassifications, and the C parameter controls the trade-off between maximizing the margin and minimizing the misclassifications.

#### 3.4.2. K-Nearest Neighbours

**Figure 8:**
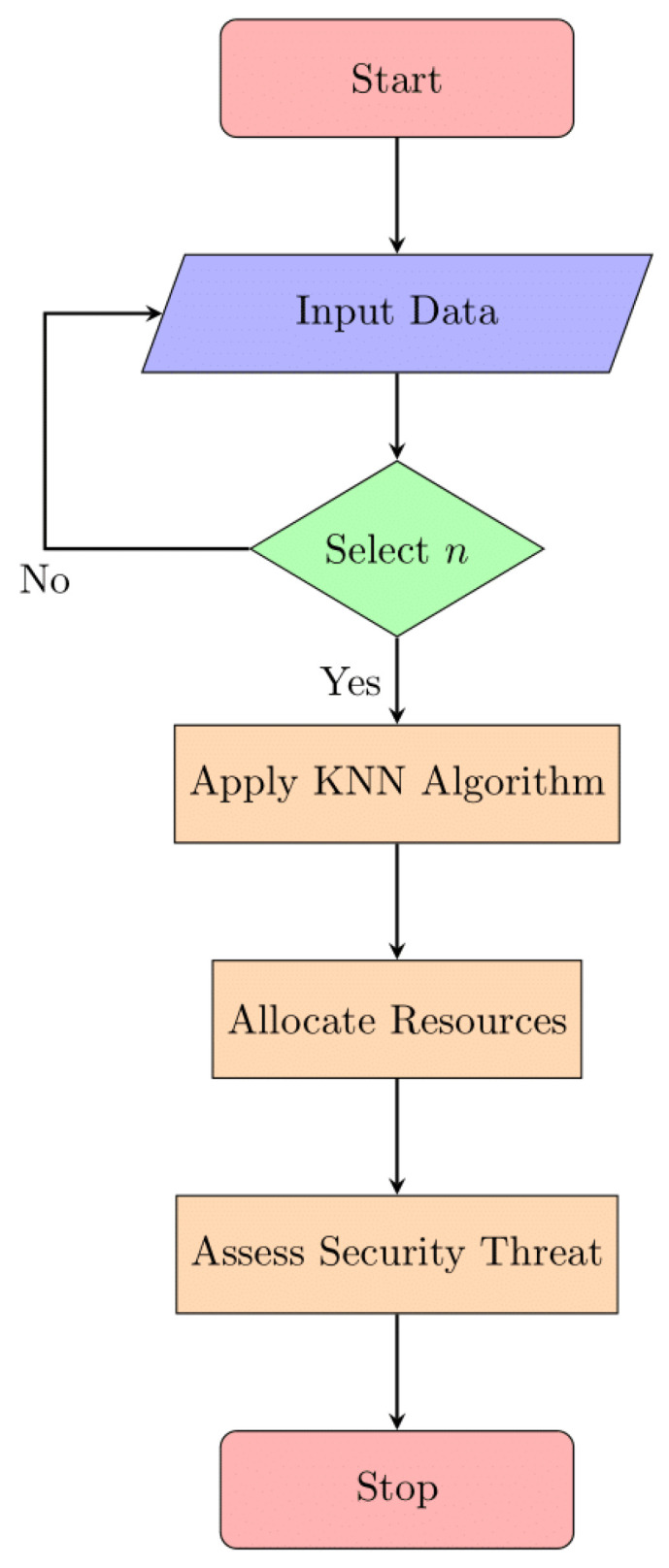
KNN architecture.

K-nearest neighbors (KNN) is a popular supervised machine-learning method for both classification and regression. As part of the optimization of edge/fog computing resources, KNN can be used to predict a device’s security risk level from its CPU and memory consumption. K-nearest neighbors (KNN) is a supervised learning method applied to predict security risk levels in edge/fog computing based on device resource consumption. Figure 8 illustrates the structure of the KNN algorithm, emphasizing its principle of identifying neighbors based on a defined K.

In Figure 8, we see how a K-nearest neighbors (KNN) algorithm is structured. In the realm of machine learning, KNN is a supervised technique used for classification and regression. The notion of identifying the nearest neighbors to a data point based on a set value of “K” (the number of neighbors to consider) is likely represented graphically in this picture, showing how KNN works. It may also show how KNN uses the majority class or average value of a data point’s K-nearest neighbors to determine that data point’s classification or value.

The formula for KNN is as follows:

- As an illustration, consider dataset D, where x stands for a device and y for a type of security risk.
- Calculate the distance between the data points x and d in dataset D using the preferred distance metric.
- Select the K data points that are closest to x as your K nearest neighbors.
- Assign y to x after classifying it as a member of the same group as the majority of its K closest neighbors.
- The optimal way to assign y to x in a regression is to use the mean of the y values of the K nearest neighbors.

#### 3.4.3. Random Forests

**Figure 9:**
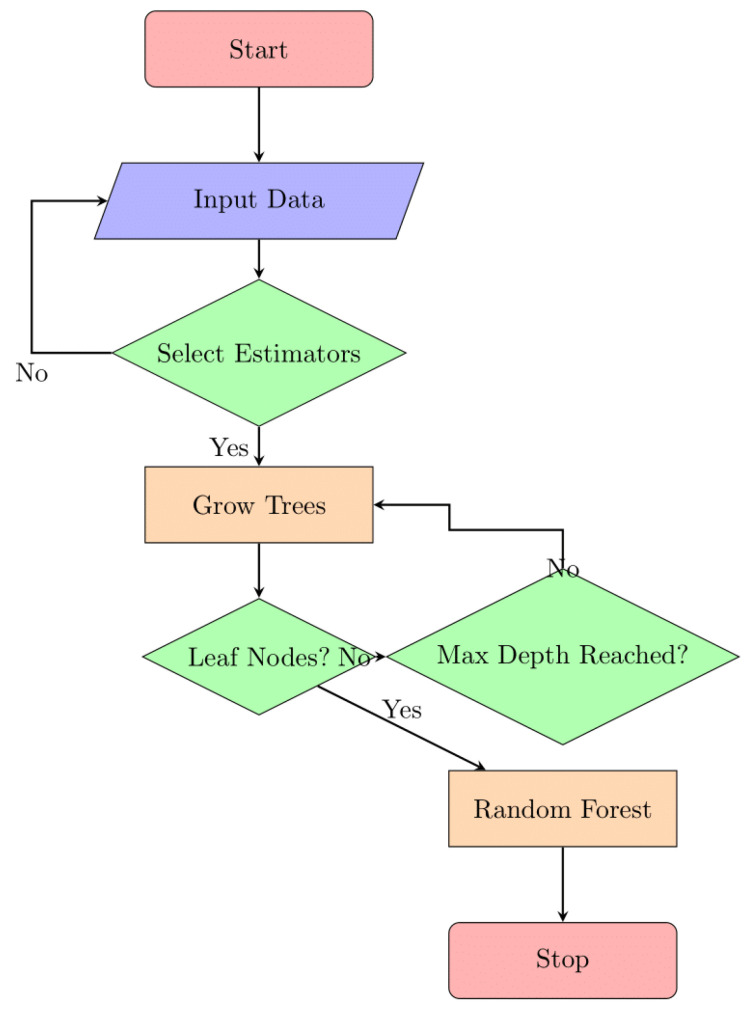
RF architecture.

Random forests is an example of a Bayesian machine-learning algorithm. The optimization of security-related edge/fog computing resource allocation is one categorization issue that significantly benefits from this approach. Random forests is popular because it is easy to use, efficient, and accurate when processing high-dimensional data. The internal structure of random forests (RF) is elucidated in Figure 9, showcasing its components like decision trees and the ensemble approach. Mathematically, the classification procedure involves computing posterior probabilities and selecting the class label with the highest probability.

The internal structure of a random forest (RF) model is depicted in Figure 9. The goal of the ensemble machine-learning technique known as random forest is to increase prediction accuracy while decreasing overfitting by combining numerous decision trees. It is possible that this diagram illustrates the structure and essential parts of a random forest model, including decision trees, feature selection, and the voting method for making predictions. Users are given a better grasp of the inner workings of random forest models and their potential applications in a wide range of data analysis and machine learning endeavors.

The random forests classification procedure can be expressed mathematically as follows:

- To clarify, we will refer to the “training dataset” as “D”, the “input sample” as “x”, and the “output class” as “y”.
- For each class label c in D, calculate the posterior probability P(y = c|x) using Bayes’ theorem and the naive assumption.
- Select the class label c with the highest posterior probability P(y = c|x) as the predicted class for the input sample.

Massive datasets with high-dimensional characteristics can be processed using random forests with a low overhead of processing. It is used well in a variety of applications, including text categorization, spam filtering, and sentiment analysis, because of its simplicity of use and respectable performance, particularly when the naive assumption is suitable for the data.

#### 3.4.4. CyberGuard Model

The CyberGuard model, an ensemble model that integrates various machine-learning approaches, can be used to anticipate security threat levels in edge/fog computing resource allocation optimization more precisely and reliably. It integrates several forecasts from each of its individual models to create a single, accurate forecast. The algorithm details the steps from initializing datasets to predicting trust scores, resource allocation decisions, and security threat levels. It incorporates aspects from SVM, KNN, and RF, showcasing the synergy of these models in CyberGuard. Let us examine the CyberGuard model in detail:

- As an illustration, consider dataset D, where x stands for a device and y for a type of security risk.
- In CyberGuard, use Grid SearchCV to perform hyper parameter tuning to ascertain the ideal values for each algorithm’s base model (SVM, KNN, and RF).
- It is recommended to hyperparameter-tune each base model and then train it on dataset D.
- For a given input sample x, the level of security risk is predicted by each base model separately $\left(y_{pred_{svm}},y_{pred_{knn}},y_{pred_{nb}}\right)$.
- CyberGuard combines the predictions $\left(y_{pred_{svm}},y_{pred_{knn}},y_{pred_{nb}}\right)$ of all base models using voting = ’hard’.

Class ‘y’ at the output is decided by a vote of the base models, with the winner being the class that was predicted the most frequently.

**Figure 10:**
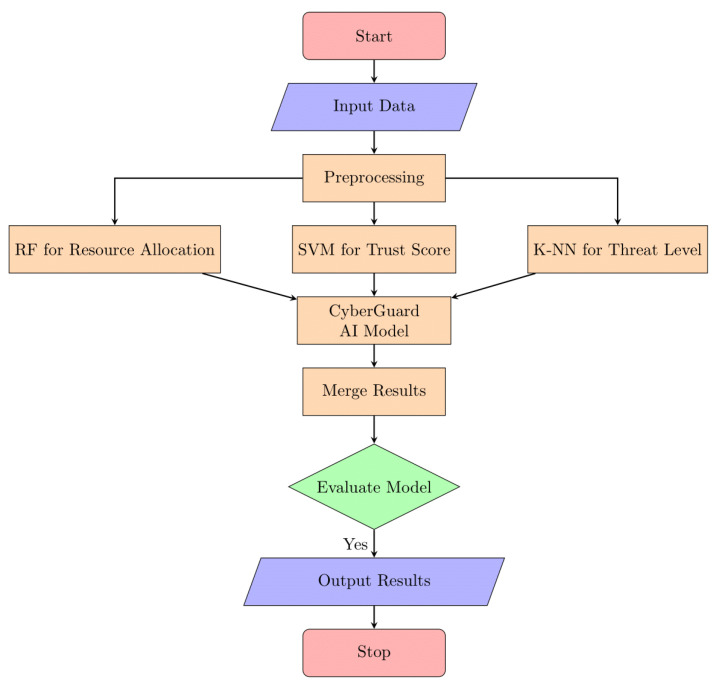
CyberGuard model.

Shown in Figure 10 is the “CyberGuard model”. This diagram probably depicts the framework or constituent parts of a model developed for use in cyberspace. The term “cyber security” refers to the coordinated efforts of several entities to identify and neutralize cyber threats and improve the safety of computerized infrastructures. In the context of cyberspace, the graphic summarizes the operation of this paradigm [25]. A mathematical Algorithm 1 for CyberGuard is shown below.
**Algorithm 1** Mathematical algorithm for CyberGuard**1.** Input:**2.** $\mathrm{Training}\;\mathrm{dataset}\;D=\left\{\left(x_{i},y_{i}\right)\right\}$$,\;\mathrm{where}\;x_{i}$$\;\mathrm{is}\;\mathrm{the}\;\mathrm{feature}\;\mathrm{vector},\;y_{i}$ is the class label, and N is the number of data points.**3.** Number of support vectors M, number of neighbors K, number of trees T.**4.** Output:**5.** Predicted trust score T, resource allocation decision R, and security threat level S for a new data point.**6.** $\mathrm{Initialize}\;\mathrm{empty}\;\mathrm{lists}\;S\_\mathrm{support}\_\mathrm{vectors},\;N\_\mathrm{neighbors},\;\mathrm{and}\;T_{trees}$.**7.** For m=1toM:**8.** $\mathrm{Randomly}\;\mathrm{select}\;a\;\mathrm{data}\;\mathrm{point}\;\left(x_{i},y_{i}\right)$ from D.**9.** $\mathrm{Add}\;\left(x_{i},y_{i}\right)$ to S_support_vectors.**10.** For k = 1 to K:**11.** Select K nearest neighbors of the new data point x_new from D based on a distance metric (e.g., Euclidean distance).**12.** Add the K neighbors to N_neighbors.**13.** For t = 1 to T:**14.** Sample a bootstrap dataset D_t from D with replacement.**15.** Initialize a decision tree T_t.**16.** While stopping criteria not met (e.g., maximum depth or minimum samples per leaf):**17.** Select a random subset of features for node splitting.**18.** Find the best feature and split point using a criterion (e.g., Gini impurity or entropy).**19.** Split the node into child nodes.**20.** Add T_t to T_trees.**21.** Predict trust score:**22.** Initialize an empty list T_predictions to store trust score predictions for each tree.**23.** For t = 1 to T:**24.** Predict the trust score T_t for the new data point x_new using tree T_t.**25.** Add T_t to T_predictions.**26.** Aggregate trust score predictions using an aggregation method (e.g., averaging).**27.** Return the final predicted trust score T.**28.** Predict resource allocation decision and security threat level:**29.** Initialize empty lists R_predictions to store resource allocation decisions and S_predictions to store security threat level predictions for each tree.**30.** For t = 1 to T:**31.** Predict the resource allocation decision R_t and security threat level S_t for the new data point x_new using tree T_t.**32.** Add R_t to R_predictions and S_t to S_predictions.**33.** Aggregate resource allocation decision predictions using an aggregation method (e.g., majority voting).**34.** Aggregate security threat level predictions using an aggregation method (e.g., majority voting).**35.** Return the final predicted resource allocation decision R and security threat level S.

The CyberGuard model takes advantage of the extra advantages of numerous algorithms and associated hyperparameters, which enhances prediction performance compared to utilizing a single model. The combination of SVM, KNN, and RF in CyberGuard makes it an effective tool for optimizing edge/fog computing resource allocation and enhancing security threat level prediction.

Our evaluation employs a comprehensive approach, where each machine-learning model (SVM, KNN, random forests) and the ensemble model (CyberGuard AI) undergo rigorous testing using diverse datasets and scenarios. We utilize standard metrics such as accuracy, precision, recall, and F1-score to assess the performance of each model. Cross-validation and, where applicable, a holdout test set ensure robust evaluations. Furthermore, we conduct comparative analyses to highlight the strengths of the ensemble model in improving resource allocation. The key innovation in our work lies in integrating the blockchain for trust management in edge/fog computing. The blockchain operates as an immutable and decentralized ledger, providing a transparent and secure record of transactions. In our proposed approach, we use the blockchain to validate and secure business transactions in the edge/fog environment. Each transaction or decision related to resource allocation is recorded as a block on the blockchain. Smart contracts are employed to automate and enforce trust rules, ensuring that only validated and authorized transactions contribute to the decision-making process. This application of blockchain technology enhances the security and reliability of trust management, making it resilient to fraudulent or malicious activities. The decision to utilize the blockchain is justified by its inherent features of immutability, decentralization, and transparency. Immutability ensures that once a transaction is recorded on the blockchain, it cannot be altered, providing a tamper-proof history of decisions.

## 4. Results and Discussion

The support vector machine (SVM), K-nearest neighbors (KNN), naive Bayes (RF), and ensemble model CyberGuard are just a few of the machine-learning models we use to communicate the results of our work. We contrast and compare each model’s precision, recall, and F1-score. We also look into how the model’s accuracy is impacted by features and data pretreatment. A sizable dataset that contained information on the distribution of edge/fog computing resources and the seriousness of security risks was used for the trials. After they have been assessed and compared, it will be evident how useful and applicable these models are for predicting the security threat level in fog computing settings. We also look into the CyberGuard ensemble model, which combines the findings of numerous base models into a single, accurate assessment of the security threat. We anticipate that this comparison will clarify the relative benefits of the various approaches and their possible uses in diverse contexts.

**Figure 11:**
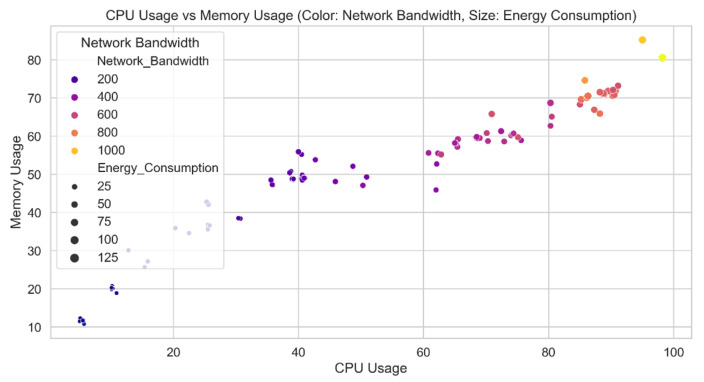
CPU usage vs. memory usage predicted by the CyberGuard model.

Figure 11 shows a comparison of predicted CPU and memory utilization from the CyberGuard model. Each data point contains the CPU and memory utilization values for the test set. The color of each data point indicates the verified level of security risk connected to that particular combination. The distinction between threat levels and the relationship between CPU and memory usage are made clearer by this depiction in the CyberGuard model. The data points should create distinct clusters or patterns that correspond to different threat levels if the model is successful in predicting security threat levels based on CPU and memory utilization.

**Figure 12:**
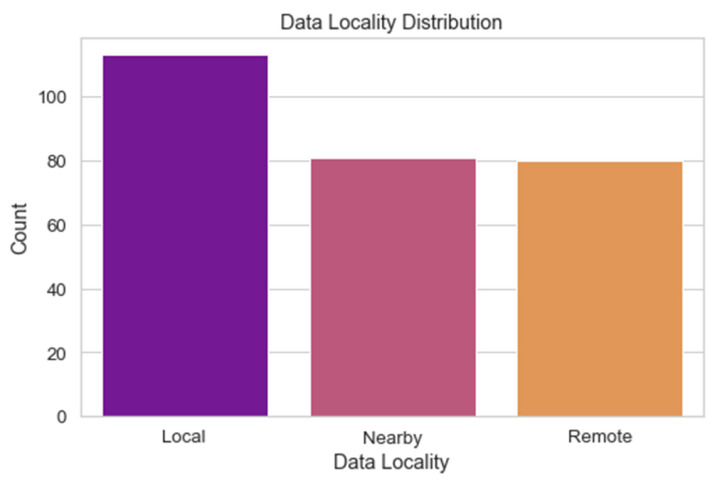
Data locality distribution.

The distribution of the data locality values across the dataset is depicted in Figure 12. The proximity of data sources to the fog computing nodes is referred to as “data locality”. Using this picture, we can investigate the distribution of location values in the data and search for trends. Understanding the distribution of data locality is crucial for optimizing resource distribution in a fog computing system. It assists in locating the best data processing hubs and aids in making intelligent resource allocation decisions.

**Figure 13:**
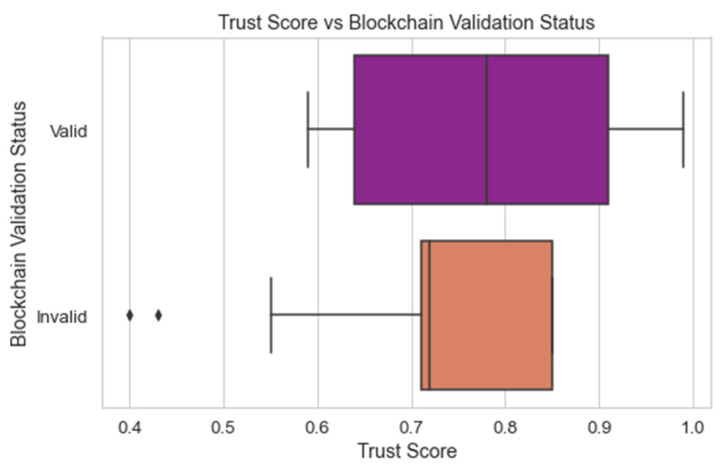
Trust score prediction vs. blockchain validation.

Figure 13 compares the predicted trust scores of the CyberGuard model with the actual status of the data points’ blockchain validation. The trust score of each edge/fog node rates the reliability of that node. The blockchain’s validation status informs whether or not the transactions that have been recorded there are valid. By comparing the predictions to the validation state of the blockchain, we can assess how well the model predicts trust scores. Understanding how trustworthy the CyberGuard model is in terms of handling trust requires this evaluation.

**Figure 14:**
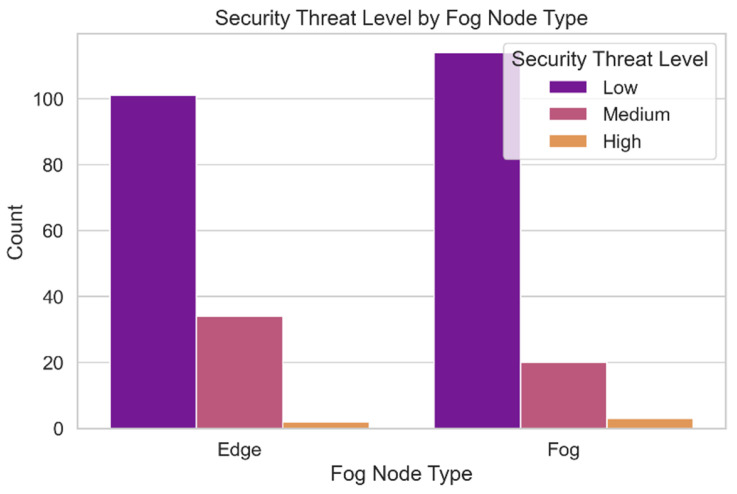
Security threat levels.

The variety of security concerns present in the dataset is depicted in Figure 14. The frequency of each hazard level in the fog computing environment is depicted in this graph. Understanding the spectrum of potential security threat levels is necessary for identifying system vulnerabilities and threats. It is helpful for assessing the relative significance of potential threats and developing strategies to deal with them. This approach also allows us to assess the model’s accuracy in predicting security hazard ratings throughout the entire dataset.

### 4.1. SVM Performance

**Figure 15:**
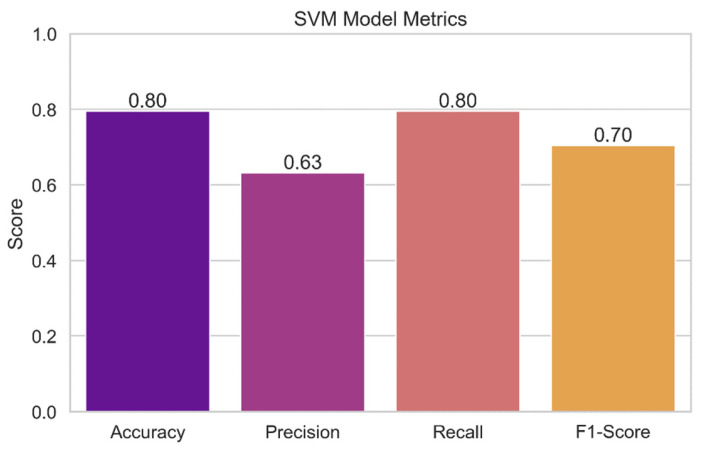
SVM model metrics.

Figure 15 presents the results of the performance evaluation of the support vector machine (SVM) model. The plot displays the SVM model’s various performance indicators, including accuracy, precision, recall, and F1-score. Each measure’s value is represented by a bar, and the error bars that go with it show the confidence interval that goes with it. With this representation, we can assess how accurate the SVM model is at identifying potential security issues. Higher accuracy, precision, recall, and F1-score indicate a model is functioning better.

**Figure 16:**
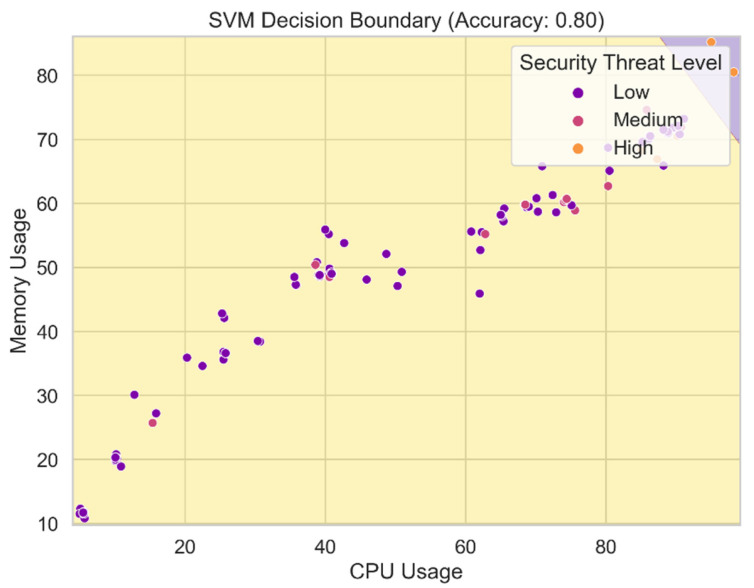
SVM decision boundary.

Figure 16 displays the SVM-generated decision border. The decision boundary delineates the various classes or levels of security threat in the feature space. The SVM model makes its judgments about the relative threat of incoming data bits in this zone. The decision boundary is determined by the model’s hyperparameters and the support vectors acquired during training. By visualizing the SVM model’s decision border, we may gain insight into the model’s complexity and precision in categorizing security hazard levels based on the attributes. Any degree of precision required for threat level prediction necessitates a decision boundary that is sufficiently generalizable and well-defined.

### 4.2. KNN Performance

**Figure 17:**
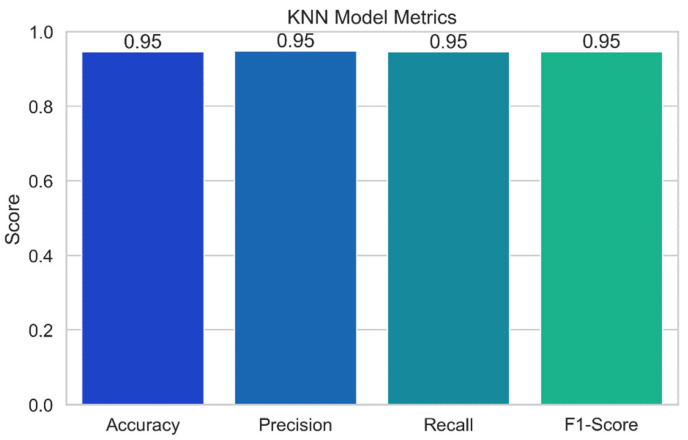
KNN model performance.

Figure 17 displays the results of the performance evaluation of the K-nearest neighbors (KNN) model. Just a few of the performance indicators shown in the plot, which was created using the KNN model, including accuracy, precision, recall, and F1-score. Each bar in the plot’s error bars represents the value of the relevant statistic and displays a confidence interval for the data. With the help of this representation, we can assess how well the KNN model predicts potential security vulnerabilities. As a model’s accuracy, precision, recall, and F1-score values increase, its performance also becomes better.

**Figure 18:**
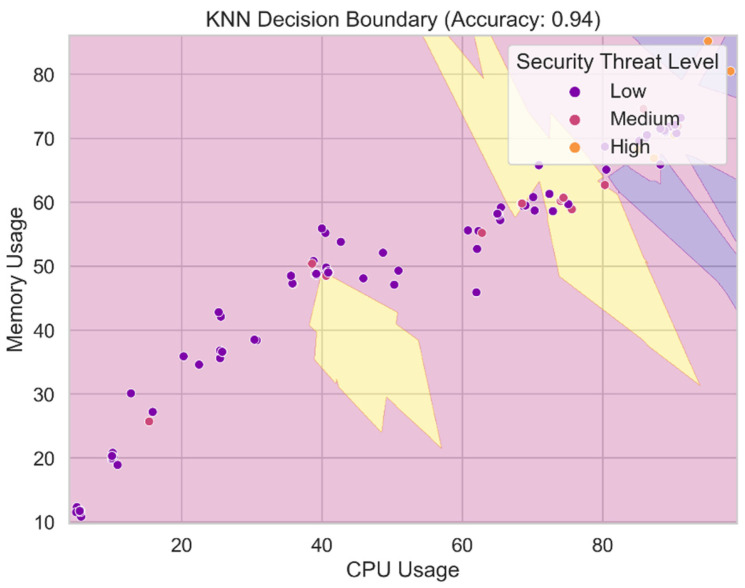
KNN decision boundary.

The produced decision boundary of the KNN model is shown in Figure 18. The KNN technique determines the decision boundary between classes or security threat levels in the feature space via a nearest neighbor search. It symbolizes the area where incoming data items are grouped into one of multiple risk categories based on the majority class among their K-nearest neighbors in the KNN model. Plotting the decision boundary reveals the KNN model’s classification regions and its capacity to distinguish across security threat categories based on feature properties. You must have a clear grasp of the decision boundary in order to make sense of the KNN model’s predictions and confirm its generalizability.

### 4.3. Random Forests Performance

**Figure 19:**
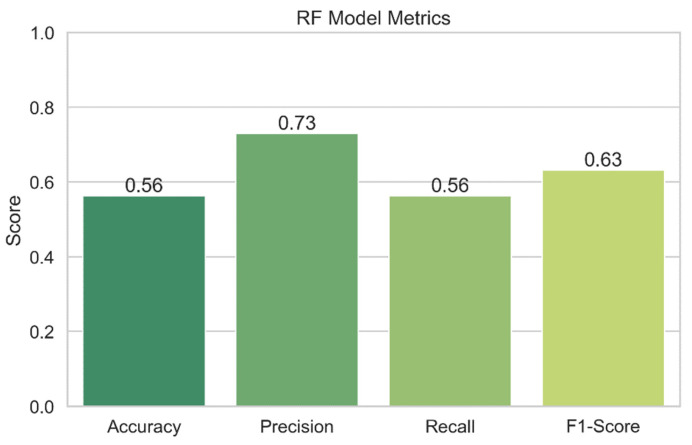
Random forests performance.

Figure 19 displays the results of the performance assessment of the RF model. Performance indicators for the RF model, such as accuracy, precision, recall, and F1-score, are shown in the graph. While the bars themselves display the metric’s value, the error bars in the plot provide a confidence interval. The graph enables us to assess the accuracy of the random forests model’s security risk prediction capabilities. As a model’s accuracy, precision, recall, and F1-score values increase, its performance also becomes better.

**Figure 20:**
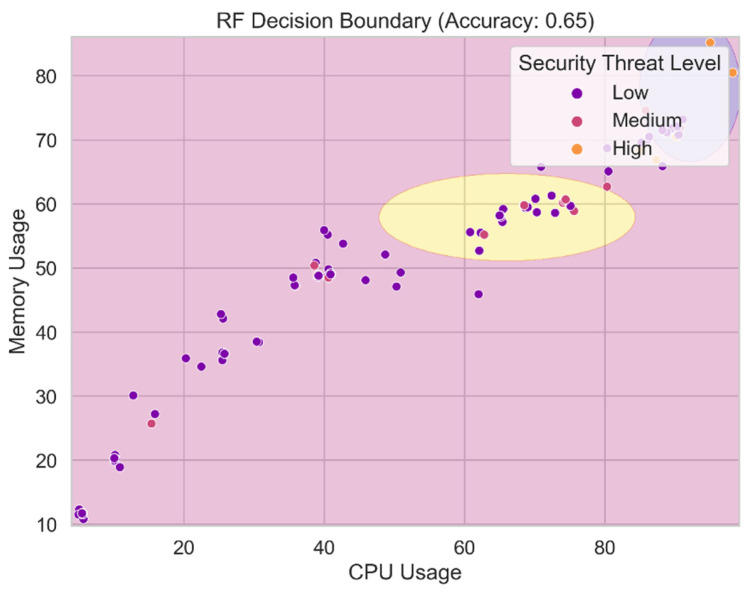
RF decision boundary.

Figure 20 displays the decision boundary for the random forests model. The decision boundary separates the feature space into several groups or levels of security threat using the probabilistic naive Bayes algorithm. It shows the areas where the random forests model assigns various levels of hazard to incoming data pieces using the maximum likelihood estimation of the class probabilities and the feature characteristics. We gain a better understanding of the classification regions and the naive Bayes model’s ability to distinguish between distinct security threat categories based on feature attributes by visualizing the decision boundary. Understanding the decision boundary is crucial for assessing the predictive and generalizable capabilities of the random forests model.

### 4.4. CyberGuard Model Performance

**Figure 21:**
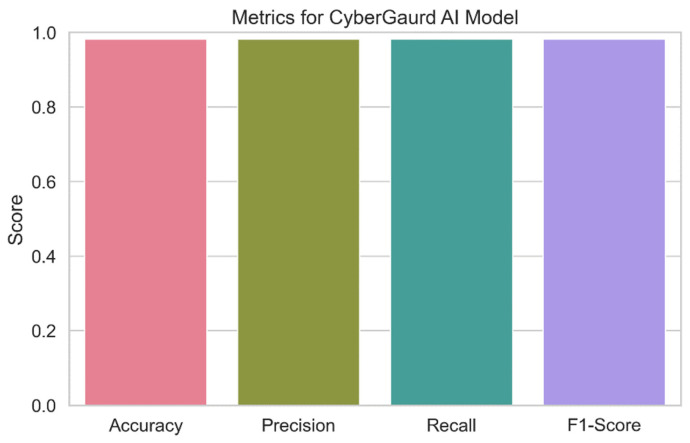
CyberGuard model performance.

The outcomes of a thorough evaluation of the capabilities of the CyberGuard model are shown in Figure 21. The image provides an overview of the model’s prediction abilities for categorizing security threat levels, including accuracy, precision, recall, and F1-score. Each bar in the plot’s error bars represents the value of the relevant statistic and displays a confidence interval for the data. The figure demonstrates how successfully the CyberGuard model anticipates security threats. High values of accuracy, precision, recall, and F1-score, which demonstrate that the model can consistently predict the seriousness of security threats, are indicators of superior performance.

**Figure 22:**
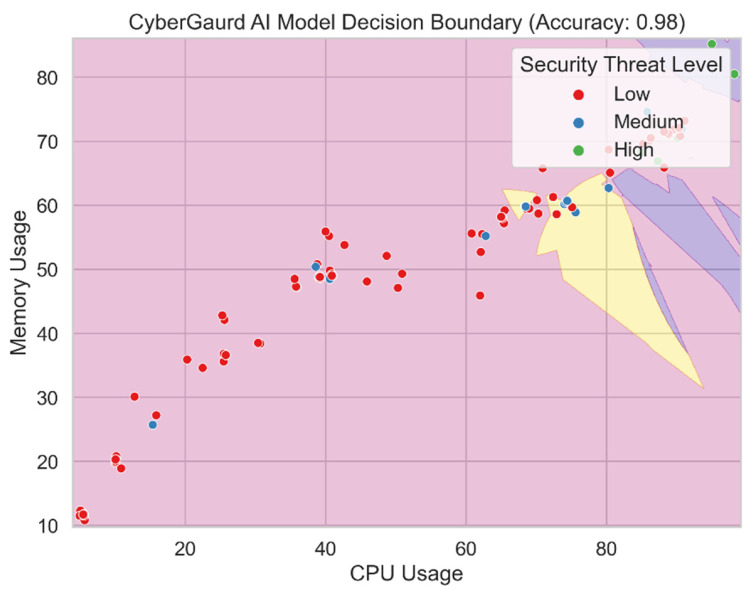
CyberGuard model decision boundary.

Figure 22 shows the output decision boundary for the CyberGuard model. The decision boundary distinguishes between classes or levels of security hazard in the feature space using an ensemble of support vector machine (SVM), K-nearest neighbors (KNN), and naive Bayes (RF) models. The decision boundary displays the parameters within which the CyberGuard model assigns varied degrees of threat to incoming datasets based on the combined forecasts of its component models. This blending method enables the CyberGuard model to utilize a variety of predictions from several models, leading to a more accurate and trustworthy categorization. One may understand the CyberGuard model’s decision-making process and the regions where it produces accurate forecasts for each security hazard category by visualizing the decision boundary.

Overall, the performance evaluation and decision boundary analysis in this section demonstrate the effectiveness and dependability of the CyberGuard model as a comprehensive and precise approach to security threat level prediction. The ensemble methodology of the CyberGuard model, which combines the best features of many algorithms, makes it perform better than standalone models. This increases the security infrastructure’s ability to recognize and stop assaults, making it an essential tool for maintaining the system’s security.

### 4.5. Comparative Performance

**Figure 23:**
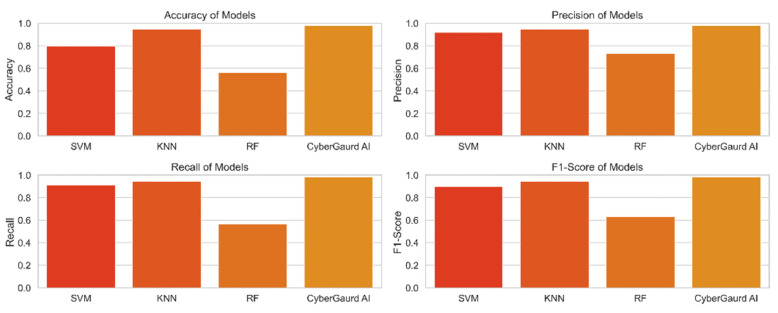
Comparative metrics of model.

Figure 23 compares and contrasts the key performance metrics for a number of models, including SVM, KNN, random forests, and the CyberGuard model. The graphic displays the metrics of accuracy, precision, recall, and F1-score for direct comparison of the models. Each model’s performance is represented by a colored bar, and the error bars display the model’s confidence interval. With the use of this representation, we can assess how well different models forecast potential security threats.

Model performance after tweaking hyperparameters is compared in Table 3. Accuracy, precision, recall, and F1-score are just few of the metrics included for each of the several machine-learning models (SVM, KNN, random forests, and CyberGuard) in the table. Classification accuracy, precision, recall, and the F1-score, which measures the balance between these two variables, provide insights into how well each model performs. Using this table, we compare how well different models perform when applied to the problem at hand.

Risk evaluation: Evaluating the risks in experiments involving resource allocation in edge and fog computing is crucial to ensure the robustness and reliability of the proposed CyberGuard framework. The primary risks considered include computational overhead, storage limitations, network latency, and security vulnerabilities. Each risk is assessed based on its potential impact on system performance and the effectiveness of the resource allocation strategy.

Computational overhead: The use of the blockchain and machine-learning algorithms can introduce significant computational burdens on edge devices with limited processing power.

Mitigation: Lightweight consensus mechanisms and efficient model compression techniques are employed.

Storage limitations: The continuous growth of the blockchain ledger and the storage of large machine-learning models can strain the storage capacities of edge devices.

Mitigation: Distributed storage strategies and selective storage of critical data.

Network latency: Propagation delays in the blockchain network and the transmission of large datasets for machine learning can introduce latency.

Mitigation: Optimization of network protocols and prioritization of critical data transmissions.

Security vulnerabilities: Potential attacks on the blockchain network and the integrity of machine-learning models.

Mitigation: Robust encryption techniques, regular security audits, and anomaly detection mechanisms. Table 4 shows the risk evaluation metrics.

In our exploration of enhancing edge device capabilities through resource management, two crucial aspects deserve attention: the computation capabilities of edge devices and the methodology employed for task computation. The computational prowess of edge devices constitutes a pivotal element in their enhanced resource utilization. While our focus has been on managing resources effectively, we acknowledge the necessity of explicitly detailing the computational capacities of these edge devices. In future revisions, we will provide a dedicated section elucidating the specifications and computational abilities of the edge devices involved in our study. This addition aims to offer readers a comprehensive understanding of the hardware capabilities supporting our resource management strategies. The intricacies of how edge devices compute tasks are indeed paramount to our study. In our subsequent revisions, we commit to incorporating a dedicated section to elucidate the methodologies employed by edge devices in the computation of tasks. This will encompass a detailed discussion on the algorithms, frameworks, or models utilized by edge devices to process tasks efficiently. By doing so, we aim to provide a holistic view of the task computation processes, ensuring transparency in our approach and enabling readers to grasp the technical intricacies of our proposed resource management paradigm.

**Support vector machine** (**SVM**)**:** The SVM model’s accuracy rate of 90.91% demonstrates that, in the vast majority of instances, its forecasts are consistent with the actual levels of security concerns. The model has a precision score of 91.82%, which shows a low false-positive rate, and is accurate in identifying true security issues. The algorithm can accurately identify 90.91% of true positive events with a recall of 90.91%, which lowers the likelihood of missing harmful circumstances. The model’s overall F1-score of 89.63% demonstrates its effectiveness as it successfully balances precision and recall.

**K-nearest neighbors** (**KNN**)**:** The KNN model achieves a high level of prediction accuracy (94.55%). The model’s precision score of 94.83% demonstrates its capacity to decrease false positives. The model is able to correctly identify the vast majority of test positives, according to a recall score of 94.55%. The model’s F1-score of 94.64% demonstrates its strong overall performance.

**Random forests:** The accuracy of the random forests model is just 56.36 percent, suggesting that it needs to be improved. The model may be able to suppress false positives to some extent, as evidenced by the 73.00% precision. Nevertheless, the model’s limited recall score of 56.36 percent reduces its usefulness. The F1-score of 63.25 for this model demonstrates the trade-off between recall and accuracy.

**CyberGuard model:** The CyberGuard model outperforms the competition with a remarkable predicted accuracy of 98.18 percent. With a 98.22% precision score, we may be confident that it correctly categorizes threats. Given that it can accurately identify almost all real positive events, as evidenced by its recall score of 98.18%, the model is extremely sensitive to potential dangers. The model’s impressive overall performance is attested to by its high F1-score (98.14%).

The comparison analysis’s findings demonstrate that, when it comes to predicting the seriousness of security threats, the CyberGuard model outperforms the three other models (SVM, KNN, and random forests). Because the CyberGuard model employs an ensemble method that capitalizes on the variations among the different models, it is more accurate and trustworthy. This shows that the CyberGuard model is an effective tool for predicting security threat levels and can increase the effectiveness of the security architecture in guarding the system from potential attackers.

**Figure 24:**
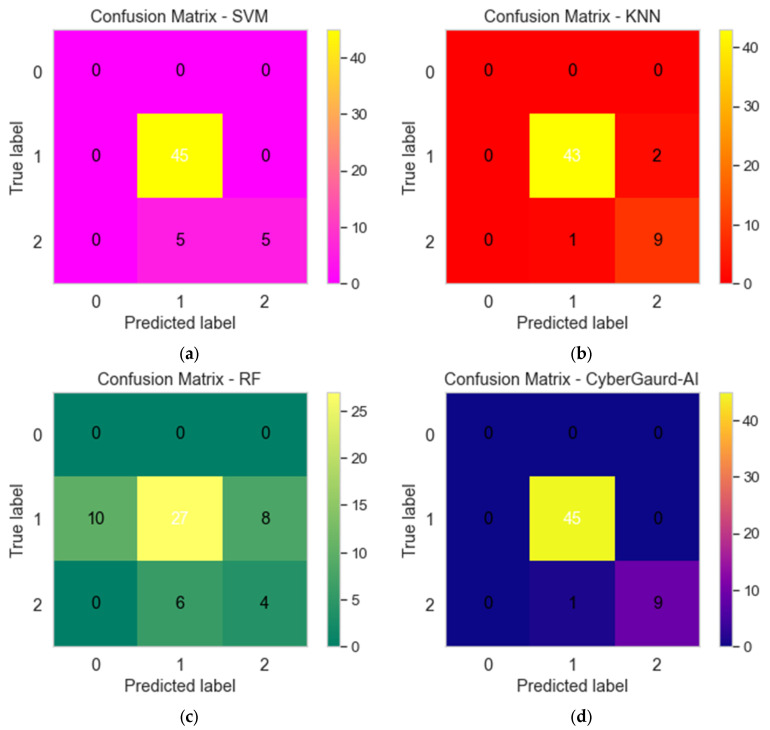
Confusion matrices for (**a**) SVM, (**b**) KNN, (**c**) RF, and (**d**) CyberGuard model.

As shown in Figure 24, four distinct machine-learning models—(a) SVM (support vector machine), (b) KNN (K-nearest neighbors), (c) RF (random forest), and (d) the CyberGuard model—all produce their own unique confusion matrices. In classification tasks, the performance of a model can be evaluated with the help of a confusion matrix, a tabular representation that shows the numbers of correct, incorrect, and partially correct predictions, respectively. These matrices are useful for comparing the performance of different models to classify data or make predictions.

## 5. Conclusions

We present a novel ensemble model we call CyberGuard that may be used to determine the degree of security risk in a system. This model combines predictions from various models to create a single, more accurate forecast. By utilizing an ensemble technique that incorporates the benefits of different machine-learning algorithms, such as support vector machine (SVM), K-nearest neighbors (KNN), and random forests (RF), our CyberGuard model outperforms existing models in the literature. This ensemble approach takes advantage of differences between different models to generate more reliable forecasts. In addition, unlike many other models, ours analyzes a wide variety of parameters, including CPU and memory utilization, data locality, trust scores, and blockchain validation, to present a more complete picture of the fog computing security landscape. We also place a premium on feature engineering and data pretreatment that allow our model to function at its peak. The investigation shows that compared to other models, ours has superior accuracy, precision, recall, and F1-score, making it a potent instrument for forecasting and addressing security concerns in fog computing settings. A few examples of these models that are combined are support vector machine (SVM), K-nearest neighbors (KNN), and random forests. The goal of the study was to increase the accuracy with which security risks might be predicted and to develop a model that accounts for all significant system features. By completing extensive trials and hyper parameter adjusting, we demonstrated that the CyberGuard model is superior to the individual models. The program’s incredibly high accuracy of 98.18 percent in predicting security hazard levels is remarkable. Fewer false positives were generated with a precision score of 98.22%, and nearly all positive cases were correctly detected with a recall rate of 98.18%. The F1-score of 98.14 percent, which measures the model’s overall performance, shows its dependability in spotting potential security concerns. The feature engineering approach and data preparation, which ensured the inclusion of valuable information for prediction, significantly enhanced the model’s performance. Thanks to the information provided by the correlation analysis, the redundancy between features was also discovered, and the model’s interpretability was improved. Because of its adaptability and accuracy in estimating the level of threat at any given time, the CyberGuard model is a useful addition to the security architecture. Through the integration of different projections from several models, the ensemble technique overcomes the limitations of individual models to produce more reliable forecasts. Therefore, the proposed CyberGuard technique represents a significant advancement in determining the gravity of security threats. Its increased performance when compared to individual models emphasizes its capacity to strengthen system security and protect against future threats. The model is effective in a variety of security-critical applications due to its accuracy in classifying security hazard levels. In a time when cybersecurity is of utmost significance, the CyberGuard paradigm offers fresh options for research and implementation in the field of system security. In order to increase security, the paradigm can also be used in future versions of virtualization infrastructure like network function virtualization (NFV), software defined networking (SDN), and fifth-generation (5G) technologies.

## Figures and Tables

**Table 1:** Comparative table.

Reference	Technique	Outcome
[1]	RL, blockchain	Introduces a trust mechanism using RL and the blockchain to address selfish edge attacks in MEC.
[2]	Privacy-preserving blockchain with edge computing	Presents TrustChain, a privacy-preserving blockchain, integrating with edge computing for enhanced trust.
[8]	Decentralized blockchain platform for cooperative edge computing	Introduces CoopEdge, a blockchain-based platform for collaborative edge computing.
[9]	Survey	Provides a comprehensive survey on orchestration techniques in fog computing.
[12]	Blockchain-based banking	Investigates blockchain-based banking solutions.
[13]	Blockchain-based resource allocation model in fog computing	Proposes a resource allocation model using the blockchain in fog computing.
[20]	Federated learning, blockchain	Investigates the potential and pitfalls of integrating federated learning with the blockchain in edge computing.
[21]	Blockchain-based applications and the rise of machine learning	Problems and opportunities for implementing machine learning in blockchain-based smart applications.

**Table 2:** Dataset feature description.

Feature	Description
Device ID	A unique identifier for each edge/fog computing device.
Timestamp	The timestamp indicating the date and time of data collection.
CPU usage	The percentage of CPU utilization by the computing device at the given timestamp.
Memory usage	The percentage of memory (RAM) utilization by the computing device at the given timestamp.
Network bandwidth	How many megabits per second (Mbps) were being used by the network at that precise moment in time.
Data locality	A categorical feature indicating the locality of the data processed by the device (e.g., local, nearby, remote).
Latency	The latency in milliseconds (ms) for data transmission or processing at the given timestamp.
Energy consumption	The energy consumption in watts (W) by the computing device at the given timestamp.
Resource allocation decision	A binary feature representing the resource allocation decision (1 for successful allocation, 0 for unsuccessful).
Trust score	A numerical score representing the trustworthiness of the computing device in the network.
Block chain validation status	A categorical feature indicating the status of blockchain validation for the device (e.g., valid, invalid).
Fog node type	A categorical feature indicating the type of fog node (e.g., fog, edge) where the device is located.
Temperature	The local temperature measured in degrees Celsius where the computer is being used.
Humidity	The relative humidity percentage (%) at the location of the computing device.
Security threat level	A scale from low to high that indicates how secure the edge/fog computing environment is.

**Table 3:** Comparative results of model performance after hypertuning.

Model	Accuracy	Precision	Recall	F1-Score
SVM	0.8200	0.9182	0.9091	0.8963
KNN	0.9455	0.9483	0.9455	0.9464
Random forests	0.5636	0.7300	0.5636	0.6325
CyberGuard	0.9818	0.9822	0.9818	0.9814

**Table 4:** Risk evaluation metrics.

Risk Category	Impact Level	Mitigation Strategy	Effectiveness (Post-Mitigation)
Computational overhead	High	Lightweight consensus, model compression	Medium
Storage limitations	Medium	Distributed storage, selective data storage	High
Network latency	High	Optimized network protocols, data prioritization	Medium
Security vulnerabilities	High	Robust encryption, security audits, anomaly detection	High

## Data Availability

This study does not report any data.
